# Cancer at the time of the COVID-19 hurricane

**DOI:** 10.1186/s13046-020-01575-1

**Published:** 2020-04-29

**Authors:** Giovanni Blandino

**Affiliations:** Oncogenomic and Epigenetic Unit, IRCCS Regina Elena National Cancer Institute, Rome, Italy

Dear Editor,

The World Health Organization (WHO) reported 18.1 million new cancer cases with 9.6 million cancer deaths in 2018. As of April 13th 2020, there are over 2.0 million cases of COVID-19 and over 100 thousands deaths from this worsening pandemic. These numbers provide testimony to the progression of two devastating “pandemic” illnesses affecting humans. While cancer is a longstanding challenge for thousands of basic, translational and clinical scientists worldwide, COVID-19 has entered the arena with an unprecedented virulence which has turned life upside down [1]. Virologists, epidemiologists, biostatisticians, immunologists, molecular biologists, modellers and infectious disease experts have joined forces to estimate the COVID-19 peak of infection, to predict its evolution, to come up with rapid and effective therapies and to prepare vaccines [2, 3]. Many of them are asked to comment on government policies, to inform authorities and to deal with the media. In that context, they must carefully weigh the statements they release to national and international communities. I believe that fighting COVID-19 under this enormous pressure that is exacerbated by the dramatic increase of deaths is extremely difficult. This might render the coordination of national and global efforts more difficult, thereby prolonging instead of shortening, the time required until we are able to defeat successful the COVID-19 pandemic. Coordinated and spontaneous fund raising is increasing worldwide; governmental agencies, private foundations, companies are launching calls for grant applications as it has never been done before. Often, deadlines or applications are within 1 month and both the review process and release of funds may be as short as a month. Unlike typical biomedical research grants, most calls for COVID-19 research projects ask for completion within 1 year, emphasizing the demand for rapid and urgent therapeutic discoveries.

Those of us who have been engaged for decades in the fight against cancer appreciate that patient stratification is critical for successful treatment. Precise cancer patient stratification also implies the identification of specific biomarkers to distinguish high and low risk subjects, to either prevent or prolong cancer insurgence, to monitor the efficacy of the treatment [4, 5]. However, the perfect cancer biomarkers have not yet been identified. One could argue that deciphering cancer, due to its high complexity, is much more difficult than SARS-CoV-2 infection which exerts its most devastating effects on the lungs. While we need to cure symptomatic COVID-19 patients, we should at the same time also study asymptomatic infected people and investigate whether any of their genetic and/or epigenetic determinants make them refractory to the emergence of clinical symptoms. Devoted and systematic biological repositories of body fluids derived from symptomatic and asymptomatic infected people will be of paramount importance for patient risk stratification and for allowing epidemiologists to exclude confounding factors and firmly identify those that drive the COVID-19 pandemic.

As cancer researchers, we should be concerned about the fact that COVID-19 is severely impacting cancer treatment and slowing down the efforts to find cures for cancer [6]. Indeed, this pandemic brought the activity of many research laboratories and oncological clinical units to a standstill. It halted patient enrolment into active clinical trials, it disabled new clinical studies and it delayed all in-person cancer meetings by almost 1 year. Consequently, cancer Centres reduced their overall patient management. This is surely the greatest problem, because cancer patients who undergo to chemo or radiotherapy are part of the fragile population that is most likely to be severely affected by SARS-CoV-2 infection and the resultant respiratory illness. “***To be or not to be***” performing COVID-19 related research activities circulates within the cancer scientific community. Each of us ponders seriously and debates passionately with colleges what to do, and whether it’s correct and, most importantly, useful to reprogram their own laboratories from cancer research to COVID-19 research activities. What to do with cancer research projects that are already funded by public and private agencies. There is no consensus on these issues. While some investigators have already re-shaped their research objectives in order to contribute to discovering COVID-19 therapeutic vulnerabilities and design vaccine production strategies, others, despite the reduced laboratory activities are continuing to pursue their own research on cancer. It’s not a matter of who is doing right or wrong; it’s time for concerted actions. COVID-19 infection might blunt social globalization. At the same time, it might frame a worldwide research platform that will not only share scientific data, but will hopefully represent also a unique occasion to act together as a global human community for both COVID-19 and cancer research.
